# Salience network resting-state functional connectivity predicts self-controlled decision-making

**DOI:** 10.1038/s41598-025-98673-x

**Published:** 2025-05-10

**Authors:** Jasmin Stein, Franziska M. Korb, Thomas Goschke, Katharina Zwosta

**Affiliations:** Faculty of Psychology, TU Dresden, Dresden, D-01069 Germany

**Keywords:** Self-control, Resting-state functional connectivity, Salience network, Human behaviour, Cognitive neuroscience

## Abstract

Salience network functional integration with the central executive network and the default mode network at rest has been shown to predict real-life self-control. It has been proposed that a network interaction index reflecting stronger functional integration of the salience network with the central executive network and reduced functional connectivity of the salience network with the default mode network represents a trait neural correlate of successful self-control exertion. Here, we attempted to replicate this result using data from our own study where 121 participants completed an fMRI self-control task comprising real-life scenarios and data from a second study (N = 79) retrieved from OpenNeuro (dataset ID: ds002643) where participants completed an fMRI food choice task. We could not replicate the proposed role of salience network resting-state functional connectivity in self-controlled decision-making in either of those data sets. Instead, we found evidence for the exact opposite effect, specifically a negative association between self-control performance and the network interaction index. The role of analysis pipelines, appropriate network ROIs, and the measurement of self-control are discussed in the context of our findings.

## Introduction

Self-control is an important prerequisite for adaptive decision-making in daily life^1,2^. Self-control has been defined as the successful alignment of behavior with personally important long-term goals. This entails the ability to resist immediate temptations when negative long-term action outcomes are expected, as well as the ability to endure an immediate aversive outcome when positive long-term action consequences are anticipated^3–6^. Failure to decide in favor of a behavioral alternative that is in line with individual long-term goals hinders goal attainment in daily life and impaired self-control has been discussed as an important transdiagnostic marker for psychiatric disorders^7,8^. Given this importance of self-control, it is unsurprising that a vast amount of research has investigated neural mechanisms underlying the successful exertion of self-control^9–12^.

In addition to the investigation of neural activation during self-controlled decision-making, another important line of research has focused on how the large-scale functional organization of the brain at rest relates to self-control performance^13–15^ or similar regulatory control functions^16^. Research investigating functional resting-state connectivity correlates traditionally focuses on a triple network model including three canonical functional networks^17^: the salience network (SN), the central executive network (CEN), and the default mode network (DMN). The SN has important nodes in the dorsal anterior cingulate cortex and the anterior insula and is important for detecting behaviorally relevant information (e.g., sensory stimuli, emotional or interoceptive information, see elsewhere^18^ for review). The SN further extends to subcortical regions involved in reward and salience processing, for instance, the amygdala and the ventral striatum^19^. The CEN is located across nodes in the lateral frontal and posterior parietal cortices^17^ and is critically involved in many higher cognitive processes such as goal-directed decision-making. The DMN contains major nodes in the posterior cingulate and medial prefrontal cortices and is thought to be involved in different types of self-referential processes^17^. It is assumed that the SN is important for switching between the activation of the CEN and the DMN and consequently guides the external or internal processing of information detected by the SN^18,20^. Specifically in the domain of cognitive control, it has been proposed that the SN detects salient stimuli from the environment and subsequently facilitates task performance by initiating and then relaying control signals to areas of the CEN, such as the dorsolateral prefrontal cortices^21^. At the same time, these control signals have been shown to deactivate the DMN^20^. In self-control research, regions of the SN and CEN have been shown to promote self-control success in line with this model: First, lower conflict-related signaling in a performance monitoring network comprising key regions typically assigned to the SN has been shown to predict lower real-life self-control performance^22^. In addition, the dorsolateral prefrontal cortex has been identified as a key region in self-control research in the past^12,23^. On the other hand, it has been argued that DMN activation is important for future-oriented or goal-directed cognition^24–26^, which itself is at the heart of self-controlled decision-making. These findings suggest that all three networks might play an important role in self-controlled decision-making and that, hence, the targeted recruitment of CEN or DMN by the SN could represent a key determinant of self-control success.

It is generally accepted that functional connectivity patterns within the nodes of each network but also between networks at rest substantially contribute to optimal cognitive functioning and that aberrancies in these patterns constitute hallmark features of psychopathology^17,27^. Hence, understanding how resting-state functional connectivity patterns relate to the successful exertion of self-control provides important insight into the neural underpinnings of self-control and potential variables to study in the context of self-control impairment.

Several studies have investigated resting-state connectivity patterns related to self-control: One notable study showed that the functional coupling of SN with CEN and DMN was predictive of desire enactment in real-life self-control conflict situations as assessed by ecological momentary assessment^13^. More specifically, the authors showed that a network interaction index computed as the difference in the strength of functional connectivity between the SN and CEN and the SN and DMN was negatively correlated with desire enactment. Hence, a stronger coupling of SN and CEN compared to SN and DMN coupling was associated with higher self-control performance. Consequently, the authors claimed that SN recruitment of CEN and suppression of DMN is vital for self-control success. The rationale here is that the observed network interaction index at rest reflects a trait propensity of the SN to integrate itself with the CEN while suppressing DMN activation^13^. While this seems like a strong assumption, there is empirical evidence showing that functional connectivity patterns at rest are reflected in neural activation during task performance^28^.

Hence, the results reported above^13^ indeed represent initial evidence for the idea that the role of SN in switching between CEN and DMN might be crucial for the successful exertion of self-control. A special role of the SN in self-controlled decision-making and cognitive control has been reported by other authors: For example, one study could show that functional coupling of the SN with a visual network was associated with lower distractibility by erotic stimuli^14^ and network dynamics within the salience network have been linked to delay discounting measures^29^. Further, in line with the results by Krönke et al.^13^, it could be demonstrated that an anticorrelation between DMN and SN at rest was associated with higher cognitive control in a study assessing cognitive control as a composite score from different cognitive tasks^30^. Next to its prominent cortical nodes, subcortical areas of the SN, including amygdala and ventral striatum, are often overlooked^19^. It is sometimes assumed from a developmental perspective that functional connectivity between these regions and the cortical networks is negatively associated with important regulatory functions^31,32^. Several studies in the domain of self-control have supported this claim: In one study, negative functional connectivity between a limbic network and the CEN was predictive of higher trait self-control and later onset of substance use in adolescence^15^. A similar finding resulted from a study showing that early substance use onset was associated with stronger functional connectivity between ventral striatum and regions of the CEN^33^. However, due to contradictory evidence in the literature and potential methodological pitfalls as discussed elsewhere^15^ these results have to be treated with caution.

Despite these previous insights into resting-state correlates of self-control, research specifically linking resting-state connectivity patterns of the salience network to successful self-control exertion remains scarce, and the relevant results are yet to be conceptually replicated. Hence, based on the findings summarized above, we aimed to investigate further the role of SN functional connectivity with CEN and DMN in self-controlled decision-making. To this end, we used two datasets from two studies: 1) Dataset one was collected by us and contains data from 121 participants who completed an fMRI self-control task using real-life decision scenarios after having undergone acute stress induction or a control protocol; 2) Dataset two (N = 79) was retreived from OpenNeuro (dataset ID: ds002643^23,34^). These data stem from a study where participants completed an fMRI self-control task comprising food choices. Our main goal was to replicate the findings reported by Krönke et al.^13^ in these datasets. Specifically, we hypothesized that a network interaction index (NII) indicating functional integration of SN with CEN and disintegration of SN with DMN would positively predict self-controlled behavior^13^. Consequently, SN-CEN functional connectivity should positively predict self-control success, whereas SN-DMN functional connectivity should be negatively related to self-control performance. We also added amygdala and ventral striatum as subcortical nodes of the SN to our analyses. Here, we expected functional connectivity between these areas and the cortical networks, particularly the CEN, to be inversely related to self-control success.

## Methods

This study was conducted in accordance with the Declaration of Helsinki and approved by the Ethics Committee at TU Dresden (EK580122019) in advance. All participants provided informed consent at the beginning of the experiment and were reimbursed for their participation with 10 €/hour or course credit.

The analysis plan for dataset one was preregistered on the OSF (https://osf.io/yd34t). In this study, we focused on the preregistered confirmatory analyses regarding the prediction of self-control performance from static functional resting-state connectivity. The preregistered exploratory analyses related to predicting acute and chronic stress and to dynamic connectivity measures are not reported here. Deviations from the preregistered procedure are reported as such. After completing the respective preregistered analyses, we explored an alternative non-preregistered analysis pipeline, which is indicated as exploratory in this report. After analyzing dataset one, we analyzed dataset two for a further replication attempt. The analysis of dataset two was not preregistered but followed the analysis pipeline applied to dataset one.

### Sample

#### Dataset one

We initially included 127 participants who underwent a functional resting-state scan and completed an fMRI self-control task. After a quality check of the preprocessing and functional denoising procedure, one participant was excluded due to poor preprocessing quality, and five participants were excluded due to visible denoising failures. Hence, all functional connectivity analyses were conducted in a sample of 121 participants (50 male, 71 female, mean age: 22.58 years, $SD = 3.86$).

#### Dataset two

Dataset two^34^ included functional resting-state fMRI and self-control task performance data from 80 participants. After the exclusion of one participant due to excessive head motion in the scanner, a sample of 79 participants was included in our analysis pipeline (40 male and 39 female participants, mean age: 24.94 years, $SD = 4.98$).

### Exclusion criteria

#### Dataset one

We included participants who were German native speakers and between 18 and 35 years old at the time of study participation. All participants meeting the following criteria were excluded from study participation: previous experience with the Trier Social Stress Test (TSST)^35^, use of hormonal contraceptives, pregnancy, smoking ($> 5$ cigarettes/week), excessive alcohol or drug use, excessive exercising, use of medication potentially affecting hypothalamic-pituitary-adrenal axis function, diagnosed physical disease impacting the hypothalamic-pituitary-adrenal axis, current or previous diagnosis of a psychiatric disorder, body mass index $\le 16$ or $> 27$, or not fulfilling standard MRI safety criteria.

#### Dataset two

Inclusion and exclusion criteria are reported in the original study^23^. In brief, the authors included healthy participants from the Netherlands between the ages of 18 and 45 years who were successfully dieting at the time of study participation but also reported enjoying unhealthy snacks and junk food.

### Experimental procedure

#### Dataset one

All participants were screened for inclusion criteria. Upon inclusion into the study, participants were invited to a laboratory session during which a six-minute resting-state fMRI scan was completed. Participants were instructed to stay still and to keep their eyes open looking at a presented fixation cross at the center of the screen. Note that this session did not only serve the purpose of collecting resting-state data but also the familiarization with the laboratory environment.

One to several days after this session, participants were invited to a second laboratory session. In this session, participants underwent acute stress induction via the TSST^35^ or a placebo control condition^36^. Immediately after the stress or control protocol, participants completed two runs of a behavioral self-control paradigm in the fMRI scanner. At the end of this session, all participants were fully debriefed. On the day after the experimental session, all participants completed a follow-up survey containing questions regarding the items of the self-control task (see below).

*Self-control task.* The behavioral fMRI self-control task was adapted from a previous study^11^ and consisted of 214 decision trials (107 in each of two runs). In each trial, participants were presented with a real-life decision context (e.g., ‘You want to go for a run but it is freezing outside.’) for 3800 ms followed by a blank screen for 200 ms and an action suggestion (e.g., ‘Go running.’) for 3000 ms. During the time of action presentation, participants could indicate on a four-point scale (‘strong no’, ‘no’, ‘yes’, ‘strong yes’) their preference to engage in the presented action (decision value). The inter-trial interval was jittered with intervals between 2.5 and 5.5 seconds. Completing both runs of the self-control task took around 60 minutes. Items where no decision value was indicated were removed from data analyses.

Crucially, the follow-up survey contained questions corresponding to each trial of the self-control task: Participants were presented once again with each context-action pair from the self-control task in the follow-up survey. Then, for each of the presented actions, participants rated the valence of anticipated short- and long-term consequences of this action on a six-point scale ranging from very negative (- - -) to very positive (+ + +). In addition, participants could indicate whether the presented context applied to their personal life situation or not. Items indicated as not applicable were excluded from all data analyses.

These ratings allowed us to identify self-control conflicts, i.e., items where the short-term consequences of an action were rated as positive and the long-term consequences as negative, or vice versa. In combination with the indicated decision preferences, we further identified self-control failures as conflict trials where the chosen action was at odds with the anticipated long-term action consequences (i.e., indicating the preference to engage in an action with negative anticipated long-term consequences or indicating the preference to refrain from an action with positive anticipated long-term consequences). Items where no short- or long-term consequence rating was provided were removed from data analyses.

For the self-control task data, we applied the following task-based exclusion criteria to ensure behavioral data quality: We excluded participants with more than 54 ($\sim$25%) missing trials, i.e., trials where no decision value was indicated during the fMRI task. We further excluded participants with less than 10 overall identified self-control conflicts or less than 10 self-control failures overall. Finally, we excluded participants with less than five identified resist temptation (positive short- and negative long-term consequences) or less than five identified endure aversion (negative short- and positive long-term consequences) conflicts. Note that this procedure deviates from the preregistered one. However, we decided to choose this procedure in line with a previous analysis of this task^37^, hence keeping our analysis strategy consistent across analyses applied to this dataset. Applying these criteria, we wanted to make sure to only include participants who actively engaged in the task and avoid biased self-control performance measures resulting from an overall low number of conflicts or failures.

*FMRI data collection.* We collected a six-minute resting-state fMRI scan for each participant. Due to a technical upgrade at our facilities, the data were collected using a Siemens 3 T Trio Trim scanner initially and a Siemens 3 T Prisma scanner after the upgrade. Functional resting-state imaging was completed using an EPI sequence with the following parameters: $TE = 25\,ms$, $TR = 2000\,ms$, flip angle $= 78^\circ$, voxel size = $3\times 3\times 3.2\,mm$. For each subject, we also collected an anatomical T1-weighted scan with the following parameters: 1) Trio Trim: $TE = 2.19\,ms$, $TR = 2400\,ms$, flip angle $= 8^\circ$, voxel size = $0.85\,mm$ isotropic; 2) Prisma: $TE = 2.20\,ms$, $TR = 2400\,ms$, flip angle $= 8^\circ$, voxel size = $0.85\,mm$ isotropic.

#### Dataset two

The experimental procedure is reported in detail by the original authors^23^. Here, we focus on a brief description of the self-control task employed by the authors. In brief, participants completed a self-control task consisting of three parts: In parts one and two, respectively, participants rated the taste and healthiness of visually presented food items. In part three, for each participant, a neutral reference food item was chosen that had been rated as neutral in health and taste during parts one and two. In each trial of part three, participants then had to decide between a presented food item from the item pool and the reference food. Participants were told that they would have to eat the chosen food from one randomly selected trial at the end of the experiment. In analogy to dataset one, we identified all self-control conflicts in each subject. Self-control conflicts were defined as trials where the presented food item was rated as tasty but unhealthy (resist temptation conflict) or as unpleasant but healthy (endure aversion conflict). We further identified self-control failures as conflict trials where participants made choices contradicting the explicit goal to make healthy food choices (i.e., choosing a food item rated as tasty but unhealthy or rejecting a food item rated as unpleasant but healthy) and self-control success as the reverse scenario.

*FMRI data collection.* The original authors^23^ collected an eight-minute resting-state fMRI scan using a 3 T Philips Achieva scanner. The following scanning parameters were used: $TE = 28\,ms$, $TR = 2000\,ms$, flip angle $= 76.1^\circ$, voxel size = $3\times 3\times 3.3\,mm$. T1-weighted images with the following parameters were also collected: $TE = 3.73\,ms$, $TR = 8.2\,ms$, flip angle $= 8^\circ$, voxel size = $1\,mm$ isotropic.

### Data analyses

#### Behavioral data

For each subject of dataset one, we determined the individual percentage of self-control success by computing $1 -$(*N* self-control failure $\div$ *N* self-control conflict). In dataset two, following the procedure described in the original study, self-control success was also computed as the relative percentage of self-control success trials compared to self-control conflict trials.

#### FMRI data preprocessing

*Dataset one.* FMRI images were subjected to a quality check (using MRIQC^38^, version 22.0.6). After visual inspection of the resulting quality metrics, we used FMRIPrep^39^ (version 20.2.7) for fMRI data preprocessing. We did not run Freesurfer’s surface reconstruction, used fieldmap-based distortion correction, and sampled our data to the MNI152NLin2009cAsym output space. All magnitude files used to compute fieldmaps were subjected to FSL’s brain extraction tool before data were entered into the preprocessing pipeline. After running FMRIPrep, we checked for participants with excessive head motion (mean $FD > 0.5\,mm$). No participant reached this value. We also conducted visual inspections for preprocessing quality.

Further processing steps were conducted using the CONN toolbox^40^ (version 22.a): First, functional data were smoothed using an 8 mm smoothing kernel corresponding to the default setting of the toolbox (note that according to the default settings of the CONN toolbox, all connectivity analyses including binary ROI masks were computed using time series from unsmoothed data and analyses using weighted component time series were conducted from smoothed functional data). Subsequently, functional data were entered into CONN’s default denoising pipeline. The following nuisance variables were used for denoising: signal from white matter and cerebrospinal fluid (5 components each, obtained via CONN’s aCompCor), estimated head motion parameters and first order derivatives (12 head motion parameters in total), outlier scans (scrubbing regressors according to the individual number of outlier scans detected for each subject by CONN’s default ART procedure). Finally, we included session and task effects and their first order derivatives (2 factors), and linear trends (2 factors) within each functional run according to CONN’s default settings. Note that we did not use global signal regression in line with CONN’s default settings and debates around this practice in functional resting-state connectivity analyses^41^. Denoising quality was visually assessed by inspecting the distribution of voxel-to-voxel connectivity. Finally, the default bandpass filter of the CONN toolbox was applied (0.008 to 0.9 Hz). In total, 121 subjects were entered into all functional connectivity analysis pipelines.

*Dataset two.* Imaging data were loaded into the CONN toolbox (version 22.a) and preprocessed using the toolbox’s default preprocessing pipeline. Then, the functional images were denoised and bandpass filtered as described above. All remaining 79 subjects (one excluded due to excessive head motion) passed visual quality inspections after denoising.

#### ROI selection

**Fig. 1:**
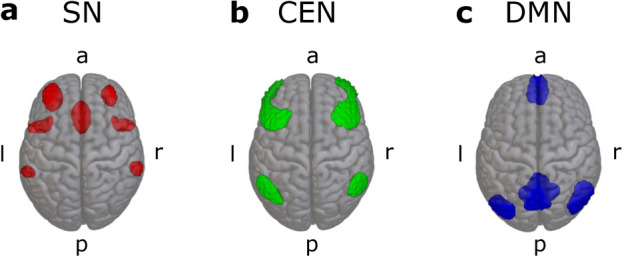
ROIs corresponding to the anatomical regions of the three networks of interest. **a**: salience network (SN), **b**: central executive network (CEN), **c**: default mode network (DMN). ROIs were created using the anatomical atlas provided by the CONN toolbox.

**Fig. 2:**
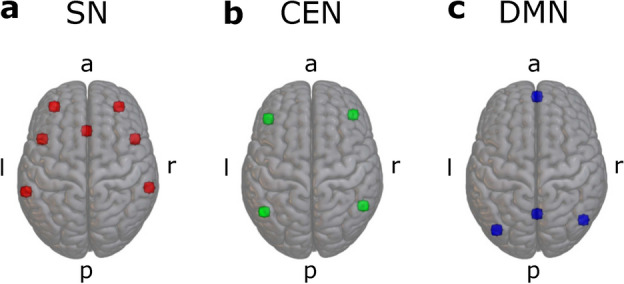
Spherical ROIs (5 mm) corresponding to the three networks of interest. **a**: salience network (SN), **b**: central executive network (CEN), **c**: default mode network (DMN). Table 1 shows the location of the center coordinates of all spheres.

**Fig. 3:**
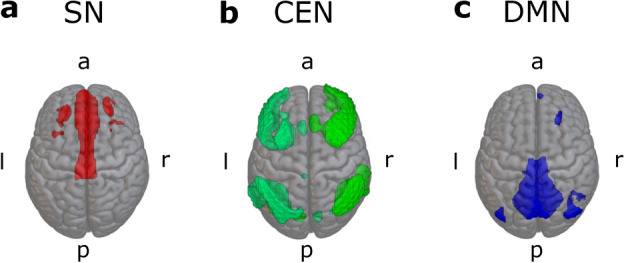
Components resulting from group-level ICA with 20 components in dataset one. **a**: salience network, **b**: central executive network (lime green = right CEN, mint green = left CEN), **c**: default mode network. Component maps were thresholded at an intensity threshold value of two.

**Fig. 4:**
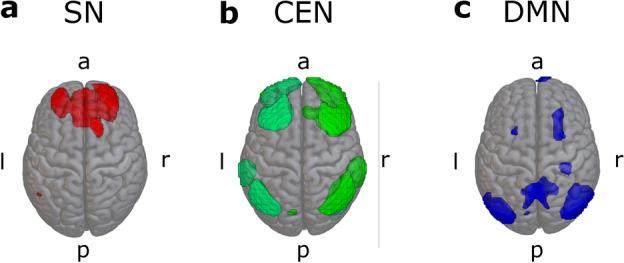
Components resulting from group-level ICA with 25 components in dataset two. **a**: salience network, **b**: central executive network (lime green = right CEN, mint green = left CEN), **c**: default mode network. Component maps were thresholded at an intensity threshold value of two.

As preregistered, a priori ROI masks constituting anatomical brain areas corresponding to the full CEN, SN, and DMN were adopted from the CONN toolbox (version 22.a) which provides network masks derived from the Human Connectome Project. For each network, we combined the masks provided by CONN into one single binary mask for each cortical network. These anatomical network ROIs are shown in Fig. 1. All 3D brain images (Figs. 1, 2, 3, 4) were created using MRIcroGL version v1.2.20220720 (https://www.nitrc.org/projects/mricrogl). For amygdala and ventral striatum, we used ROI masks from the standard atlas provided by the CONN toolbox. We combined left and right amygdala (AMY) and nucleus accumbens (ventral striatum, VS) masks, respectively, to create bilateral masks.

In addition, as preregistered, we computed group-level ICA analyses (see below) for generating data-driven ROIs corresponding to our networks of interest. Components resulting from these group-level ICA analyses were thresholded at a value of two and binarized into one ROI mask corresponding to each component of interest.

**Table 1:** Center coordinates used to create 5 mm spheres corresponding to nodes of the salience, central executive, and default mode network. ACC = anterior cingulate cortex, AI = anterior insula, RPFC = rostral prefrontal cortex, SMG = supramarginal gyrus, LPFC = lateral prefrontal cortex, PPC = posterior parietal cortex, MPFC = medial prefrontal cortex, LP = lateral parietal cortex, PCC = posterior cingulate cortex.

**Label**	**Center [x, y, z]**
**Salience network**	
ACC	[0,22,35]
AI L	[-44,13,1]
AI R	[47,14,0]
RPFC L	[-32,45,27]
RPFC R	[32,46,27]
SMG L	[-60,-39,31]
SMG R	[62,-35,32]
**Central executive network**	
LPFC L	[-43,33,28]
LPFC R	[41,38,30]
PPC L	[-46,-58,49]
PPC R	[52,-52,45]
**Default mode network**	
MPFC	[1,55,-3]
LP L	[-39,-77,33]
LP R	[47,-67,29]
PCC	[1,-61,38]

In an exploratory, non-preregistered, alternative analyses pipeline, we recreated the ROIs used by Krönke et al.^13^: we created 5 mm spheres around the seed coordinates provided by CONN corresponding to each node of each of the cortical networks of interest. This resulted in seven ROIs corresponding to the SN, four ROIs corresponding to the DMN, and four ROIs corresponding to the CEN. These ROIs are shown in Fig. 2 and the center coordinates used to create the spheres are shown in Table 1. The rationale behind this analysis was the attempt to replicate the results reported by the original authors^13^ as closely as possible. We also did not combine left and right amygdala and ventral striatum into bilateral masks here.

In an additional exploratory approach, we computed weighted sum time series from components of interest identified during group-level ICA instead of binary component masks. This approach was taken to explore an alternative method for computing functional connectivity measures from group-level ICA results.

#### FMRI data analyses

To analyze resting-state functional connectivity, we used the CONN toolbox^40^ (version 22.a). We computed the following ROI-to-ROI functional connectivity analyses in each of the two datasets:

*ROI-to-ROI functional connectivity analysis using anatomical ROIs.* We computed ROI-to-ROI functional connectivity as bivariate correlations between source time series extracted from the above-described anatomical network ROIs. For each subject, standardized correlation coefficients indicating functional connectivity between SN and CEN, SN and DMN, CEN and amygdala, CEN and ventral striatum, DMN and amygdala, and DMN and ventral striatum were extracted. More specifically, functional connectivity strength was represented by Fisher-transformed bivariate correlation coefficients from a general linear model, estimated separately for each pair of ROIs, characterizing the association between their BOLD signal time series. Individual scans were weighted by a boxcar signal spanning the full duration of the scan convolved with an SPM canonical hemodynamic response function and rectified. Group-level analyses were performed using a General Linear Model. For each individual connection a separate GLM was estimated, with first-level connectivity measures at this connection as dependent variables (one independent sample per subject). Connection-level hypotheses at the group-level were evaluated using one-sample *t*-tests. Inferences were performed at the level of individual functional connections and the results were thresholded using a familywise corrected p-FDR < .05 (Benjamini-Hochberg method) connection-level threshold.

In the exploratory alternative analysis pipeline, this analysis was conducted using the individual spherical ROIs instead of the combined binarized network ROIs.

Finally, we computed a network interaction index (NII) as suggested by Krönke et al.^13^. This index was computed as the difference between the correlation coefficients indicating functional connectivity between SN and CEN and between SN and DMN. The rationale behind this index is the assumption that the SN plays an integral role in switching between CEN and DMN^17^. NII is thus a measure of SN integration with CEN and disintegration with DMN^13^.

*ROI-to-ROI connectivity analysis using ICA-based ROIs.* We repeated the static connectivity analysis outlined above using a data-driven group-level approach to identify the networks of interest: We computed the preregistered group-level ICA to identify 30 (in line with Krönke et al.^13^) data-driven functional networks in our sample. Subsequently, we used CONN to compute correlations between all 30 components and the anatomical ROIs corresponding to CEN, DMN, and SN from CONN’s atlas. However, this approach did not yield satisfactory component solutions representing the networks of interest: In dataset one, group-level ICA with 30 components only yielded a right hemispheric CEN and no corresponding contralateral component could be identified. In dataset two, no satisfactory SN component could be identified. Additionally, our alternative preregistered approach to prespecify only six components did also not yield satisfactory component solutions. The predefinition of a fixed number of components represents a challenge in neuroimaging research using ICA to date. Since it was important to us to detect ICA components reflecting our anatomical networks of interest, we deviated from the preregistered procedure and computed several ICAs with 20, 25, 35, and 40 components (following a similar approach used in past research^42^) respectively in each dataset and chose the component solution yielding satisfactory components corresponding to all networks of interest in each dataset. For dataset one, the best solution resulted from the 20-component ICA, in dataset two the 25-component solution provided the best matches to our anatomical data. The match between anatomical ROIs and ICA components was determined based on correlation coefficients and visual inspection. The components with the highest correspondence to the anatomical ROIs were saved as binarized ROI masks (thresholded at an intensity value of two) and entered into a ROI-to-ROI connectivity analysis following the steps described above. In the alternative analysis pipeline, we computed weighted sum time series from the components of interests and entered these into the ROI-to-ROI connectivity pipeline instead of binarized masks. For amygdala and ventral striatum, we used the anatomical masks described above.

For all analysis pipelines where multiple ROIs belonged to the same cortical network (i.e., for spherical ROIs and ICA components), we also averaged functional connectivity measures between all pairwise combinations of ROIs from each network pair of interest to compute average between-network connectivity. The resulting values were then used to calculate NII as the difference between average SN-CEN and SN-DMN functional connectivity. We tested average between-network functional connectivities for statistical significance using one-sample *t*-tests. Here, we conducted FDR-correction at the level of individual connections for the respective number of comparisons (10 comparisons for thresholded ICA components, 21 comparisons for spheres and weighted components) using the Benjamini-Hochberg method and an inference criterion of *p*-FDR < .05. Finally, for the joint analysis of both datasets, we proceeded similarly, hence computed averaged connectivity values across all participants of both datasets and applied the respective FDR-correction.

#### Predicting self-control performance

Using the extracted functional connectivity measures, we computed three linear regression models to predict self-control performance in each dataset using MATLAB’s ‘fitlm’ function which employs an Iteratively Reweighted Least Squares procedure for model fitting. Model one included NII as a single predictor variable. In model two, self-control performance was predicted by individual bivariate correlation coefficients (computed as reported above) representing SN-CEN and SN-DMN functional connectivity, and in model three by CEN-AMY, CEN-VS, DMN-AMY, and DMN-VS functional connectivity. Note that we computed connectivity values with VS and AMY by averaging across connectivities with left and right hemispheric ROIs in the case of 5 mm spherical ROIs and weighted ICA components. In case of dataset one, stress group and its two-way interaction with all other model predictors were entered into the models to account for the two experimental groups in the study. In an exploratory analysis, we combined the data from both datasets and computed the regression models (disregarding the group factor from dataset one) in the combined dataset. Here, self-control performance was z-standardized across participants from both datasets before data analyses.

In addition to computing these preregistered regression models, we exploratively added self-control performance as a covariate to all ROI-to-ROI connectivity analyses in the CONN toolbox and tested for clusters of functional connectivity patterns significantly associated with self-control performance in each dataset. Note that here, we applied cluster- instead of connection-level inference. Cluster-level inferences were based on parametric statistics within- and between- each pair of networks, with networks identified using a complete-linkage hierarchical clustering procedure based on ROI-to-ROI anatomical proximity and functional similarity metrics. Results were thresholded using a combination of a $p <.05$ connection-level threshold and a familywise corrected *p*-FDR $<.05$ cluster-level threshold.

## Results

### Self-control performance

#### Dataset one

The mean proportion of self-control success in dataset one was .62 ($SD =.11$). Hence, participants exerted self-control successfully in the majority of conflict trials.

#### Dataset two

The mean proportion of self-control success in dataset two was .23 ($SD =.25$). Hence, self-control performance was overall low with large inter-individual variability. Only 15 out of 79 participants made self-controlled decisions in at least 50% of the trials requiring self-control (see original study^23^ for more detail on the behavioral data of this study).

### Identified ICA group-level networks

#### Dataset one

The ICA component with the strongest correlation with anatomical regions of the SN is shown in Fig. 3a. The component captured anterior cingulate cortex and rostral PFC areas typically included in the SN. The components corresponding to the right- and left-hemispheric CEN are shown in Fig. 3b. These components included dorsolateral prefrontal and posterior parietal cortices as well as smaller clusters in the temporal cortex and cerebellum. The component reflecting DMN is shown in Fig. 3c. This component included clusters spanning posterior cingulate cortex, precuneus, lateral parietal cortex, and a small cluster in medial prefrontal cortex typically considered part of the DMN. In addition, the component also included a small cluster in the right lateral prefrontal cortex.

#### Dataset two

The component showing the largest correlation with an anatomical map of the SN is depicted in Fig. 4a. The component included one large cluster of voxels comprising the cingulate cortex and rostral PFC areas of the SN. The two components with the largest correspondence to bilateral CEN are shown in Fig. 4b. Again, these components mainly included dorsolateral prefrontal and posterior parietal cortices. The DMN component is shown in Fig. 4c and contained clusters representing posterior cingulate and lateral parietal cortices. Some smaller clusters in anterior and dorsolateral prefrontal areas were also included in this component.

### Between network connectivity patterns

#### Dataset one

**Fig. 5:**
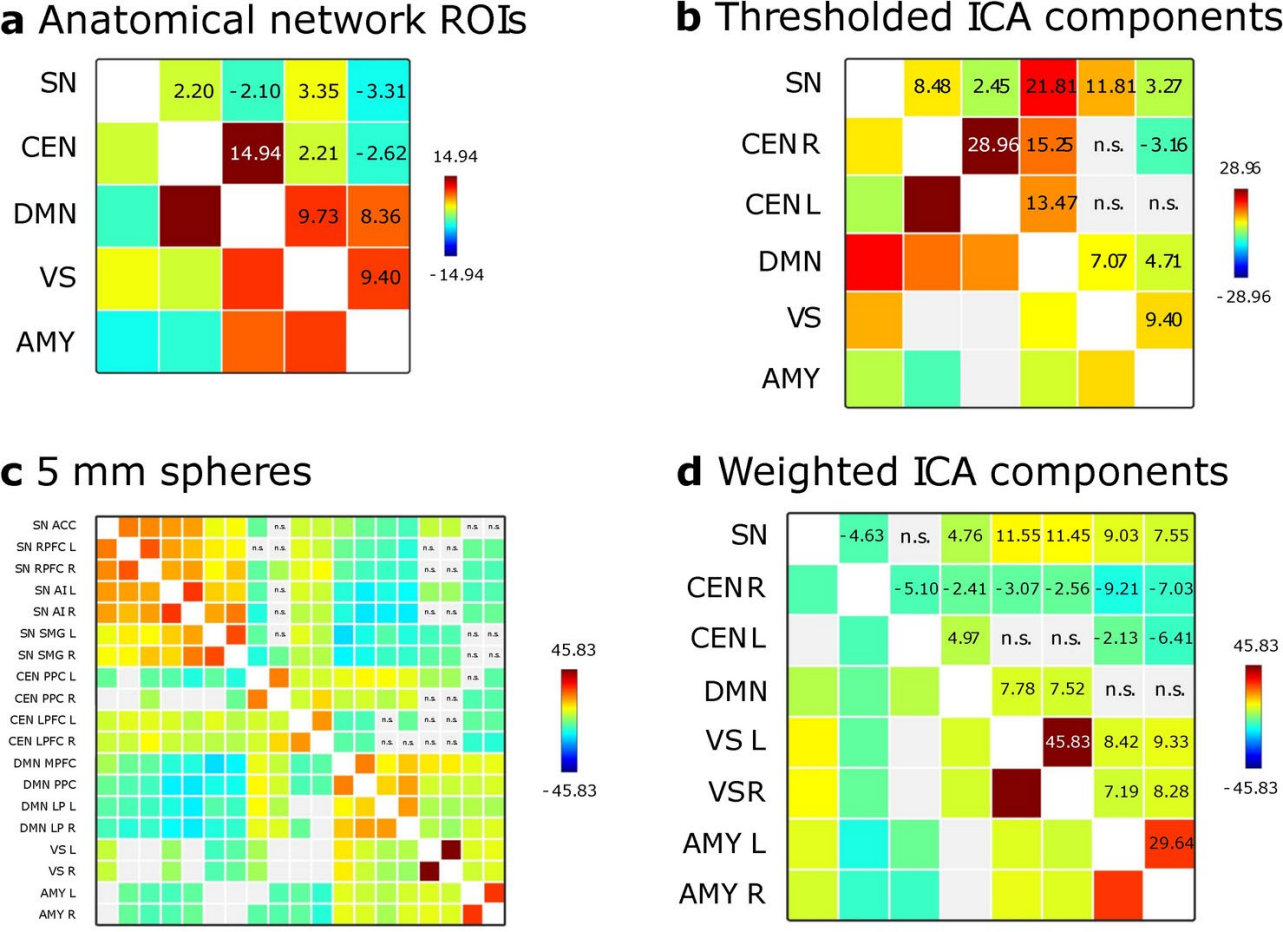
Overall between-network connectivity patterns in dataset one. **a**: functional connectivity between anatomical network ROIs, **b**: functional connectivity between network masks created from binarized group-level ICA components, **c**: functional connectivity between 5 mm spheres, **d**: functional connectivity between weighted sum time series resulting from group-level ICA. Color scale and values indicate *T*-values for statistically significant connectivities (FDR-corrected at $p <.05$).

Figure 5 shows statistically significant overall functional connectivity patterns between the identified network ROIs in dataset one (a: full anatomical networks, b: binarized ICA components, c: 5 mm spheres, d: weighted sum components). Notably, for anatomical network ROIs, functional connectivity between SN and CEN was significantly positive ($t = 2.20, p =.03$), and functional connectivity between SN and DMN was significantly negative ($t = -2.10, p =.04$) as expected^13^.

For binary ROIs from thresholded ICA components, however, SN functional connectivity was significantly positive for right and left CEN (right: $t = 8.48, p <.001$, left: $t = 2.45, p =.02$), as well as overall ($t = 6.27, p <.001$) and for DMN ($t = 21.81, p <.001$).

Using the weighted sum time series reflecting ICA components, SN functional connectivity was significantly negative with right but not left CEN (right: $t = -4.63, p <.001$, left: $t = 0.71, p =.53$), and overall SN-CEN connectivity was significantly negative ($t = -3.07, p <.01$). Further, SN showed positive functional connectivity with DMN ($t = 4.76, p <.001$).

Average between-network SN connectivity was significantly positive with CEN ($t = 4.94, p <.001$) and negative with DMN ($t = -13.34, p <.001$) when 5 mm spheres were used as network nodes.

#### Dataset two

**Fig. 6:**
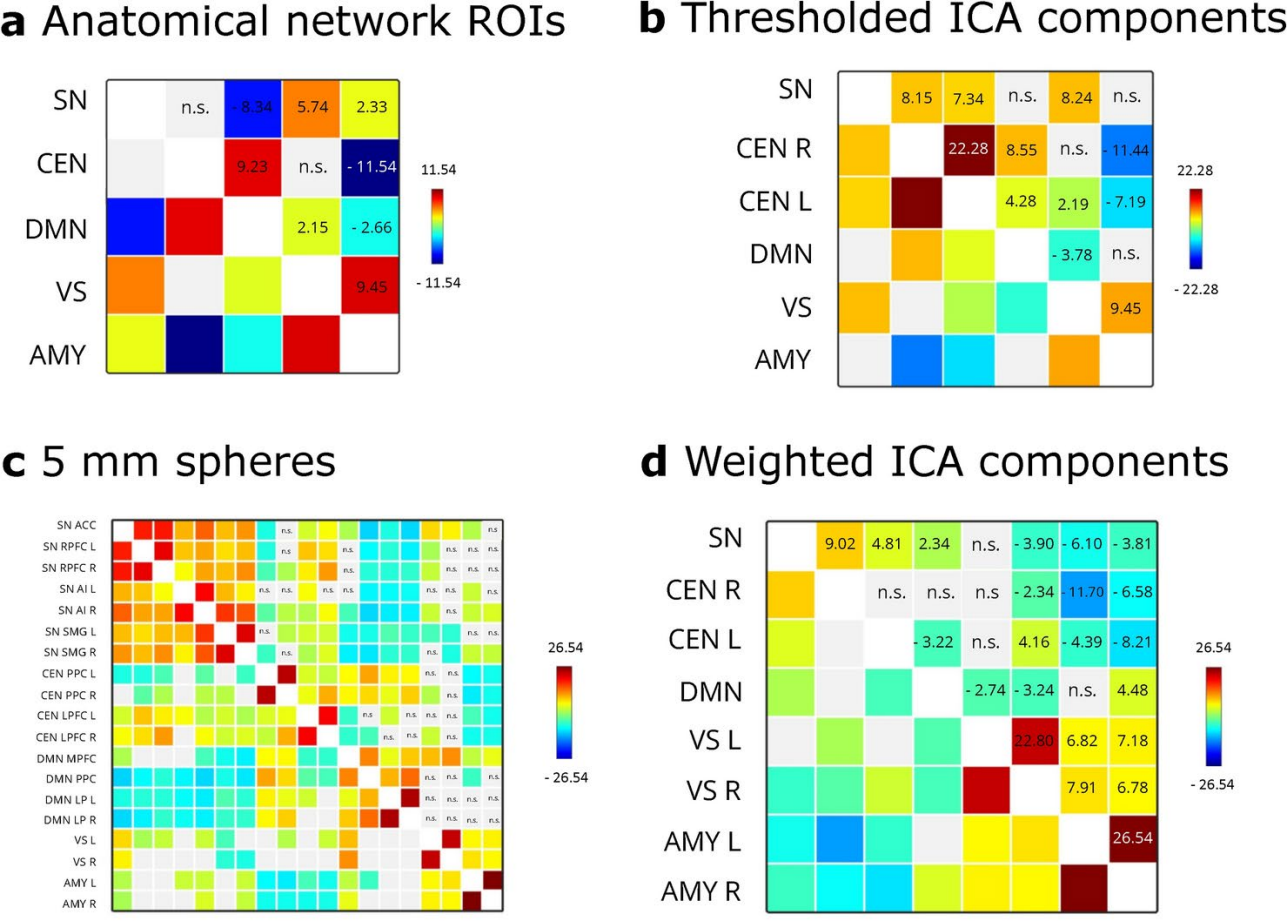
Overall between-network connectivity patterns in dataset two. **a**: functional connectivity between anatomical network ROIs, **b**: functional connectivity between network masks created from binarized group-level ICA components, **c**: Functional connectivity between 5 mm spheres, **d**: Functional connectivity between weighted sum time series resulting from group-level ICA. Color scale and values indicate *T*-values for statistically significant connectivities (FDR-corrected at $p <.05$).

Figure 6 shows significant overall functional connectivity patterns between the identified network ROIs in dataset two (a: full anatomical networks, b: binarized ICA components, c: 5 mm spheres, d: weighted sum components). For anatomical network ROIs, SN and DMN showed negative functional connectivity ($t = -8.34, p <.001$). SN-CEN functional connectivity did not reach statistical significance but was positive descriptively ($t = 0.61, p =.6$).

Using thresholded binary ROIs representing ICA components, descriptively, a similar pattern arose: SN-DMN functional connectivity was not statistically significant but descriptively negative ($t = - 0.21, p =.89$), and SN-CEN functional connectivity was significantly positive for right and left CEN (right: $t = 8.15, p <.001$; left: $t = 7.34, p <.001$) as well as overall ($t = 8.33, p <.001$).

When using the weighted sum time series to represent ICA components, functional connectivity between SN and DMN was significantly positive ($t = 2.34, p =.02$). SN-CEN functional connectivity was also significantly positive for left and right CEN (right: $t = 9.02, p <.001$; left: $t = 4.81, p <.001$) as well as overall ($t = 10.72, p <.001$).

Average between-network SN connectivity was negative with DMN ($t = -10.59, p <.001$) and positive with CEN ($t = 5.70, p <.001$) when using 5 mm spheres to represent the network nodes.

In sum, in dataset one, using anatomical ROIs (full ROIs or spheres) yielded the expected overall between-network connectivity pattern, namely positive connectivity between SN and CEN and negative connectivity between SN and DMN. In dataset two, all network specifications except for the weighted time series approach yielded the expected pattern descriptively, however, not all respective connectivity values reached statistical significance.

### Predicting self-control

#### Dataset one

**Fig. 7:**
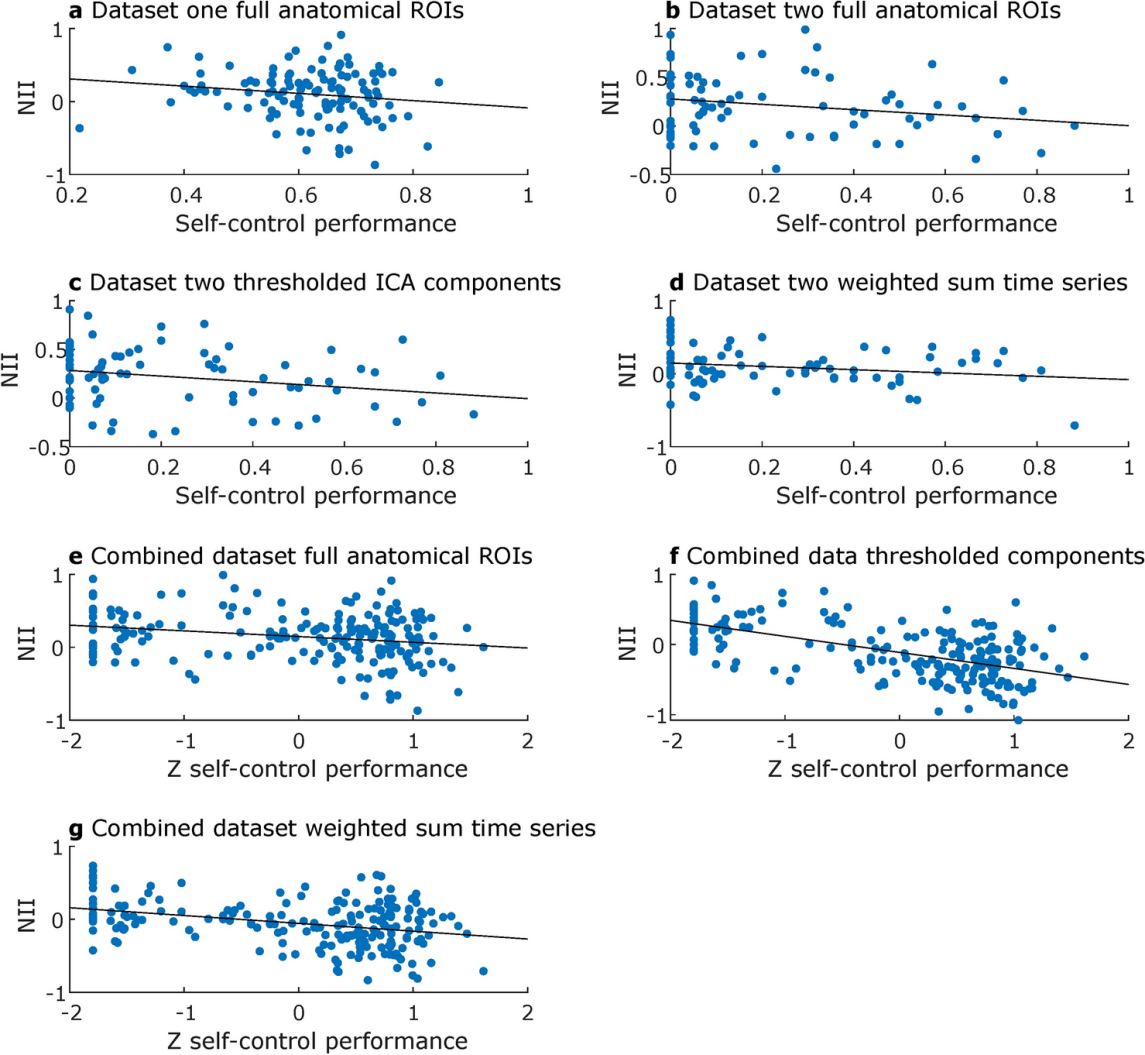
Linear association between self-control performance and the network interaction index (NII) plotted for datasets and analysis pipelines where the respective association reached statistical significance in our regression analyses. The black lines represent the least squares line for each scatter plot.

*ROI-to-ROI connectivity analysis using full anatomical networks*. We found a significantly negative effect of NII on self-control success ($\beta = -0.08, p =.03$), see Fig. 7a, directly contradicting our hypothesis. Further, there was a significantly positive effect of SN-DMN connectivity on self-control success ($\beta = 0.1, p =.04$) indicating that higher (less negative) SN-DMN functional connectivity was associated with higher rates of self-control success as opposed to our hypothesis. The model coefficient for SN-CEN functional connectivity was slightly negative ($\beta = -0.06$, $p = .18$) but did not reach statistical significance.

We did not find any statistically significant functional connectivity patterns predicting self-control performance in the analyses using thresholded component maps, 5 mm spheres, or weighted sum component time series. Further, adding behavioral self-control performance as a covariate to a second-level model specified in the CONN toolbox did not yield any statistically significant results for either of the four analysis approaches. In addition, in none of the analysis approaches, the interaction between any of the functional connectivity indices and experimental group (stress vs. control) reached statistical significance.

#### Dataset two

*ROI-to-ROI connectivity analysis using full anatomical networks*. In this analysis, again, self-control performance was negatively predicted by NII in direct opposition to our hypothesis ($\beta = - 0.18, p =.04$), see Fig. 7b. No further significant results emerged. However, descriptively, SN-CEN functional connectivity was negatively associated with self-control performance ($\beta = -0.16, p =.14$), and SN-DMN functional connectivity was positively associated with self-control performance ($\beta = 0.23, p =.08$).

*ROI-to-ROI connectivity analysis using thresholded components*. Using thresholded component maps, NII negatively predicted self-control success ($\beta = - 0.20, p =.03$), see Fig. 7c. This effect was presumably driven by a significantly positive effect of SN-DMN functional connectivity on self-control performance ($\beta = 0.24, p =.04$). SN-CEN functional connectivity showed a descriptively negative association with self-control performance ($\beta = -0.16, p =.18$).

*ROI-to-ROI connectivity analysis using 5 mm spheres*. None of the estimated regression models yielded a significant predictor of self-control success, all $p >.05$. In model one, there was a trend towards a negative relationship between NII and self-control performance ($\beta = - 0.32, p =.06$), hence in the opposite direction of our hypothesis.

*ROI-to-ROI connectivity analysis using weighted sum time series*. Using weighted time series from the group-level ICA components again yielded a significantly negative effect of NII on self-control performance ($\beta = - 0.21, p =.048$), see Fig. 7d. No other effect was statistically significant. However, testing for an effect of self-control success by adding behavioral self-control performance as a covariate to a second-level model specified in the CONN toolbox yielded a statistically significant cluster indicating that functional connectivity of left CEN with SN and with right CEN, respectively, negatively predicted self-control success, $F(3, 75) = 4.32, p =.04$, FDR-corrected at the cluster level. This negative effect of SN-CEN functional connectivity on self-control success directly contradicts our hypothesis and explains the negative effect of NII reported above.

#### Joint analysis of both datasets

In the combined dataset, overall SN-CEN functional connectivity was positive when using anatomical network ROIs ($t = 2.11, p =.045$), thresholded ICA components ($t = 10.00, p <.001$), 5 mm spheres ($t = 7.18, p <.001$), and weighted sum component time series ($t = 2.58, p =.01$). SN-DMN functional connectivity was overall negative when using full anatomical ROIs ($t = -6.05, p <.001$) or 5 mm spheres ($t = -16.44, p <.001$) and positive when using weighted sum time series ($t = 5.24, p <.001$) and thresholded component maps ($t = 11.87, p <.001$). Hence, in the full sample, the expected overall connectivity pattern was present for full anatomical ROIs and 5 mm spheres.

Using full anatomical network masks, the combined data yielded a statistically significant negative effect of NII on self-control performance ($\beta = -0.76, p <.001$) directly contradicting our hypothesis, see Fig. 7e. This effect was presumably driven by a positive effect of SN-DMN functional connectivity on self-control success ($\beta = 1.29, p <.001$). Further, functional connectivity between DMN and amygdala ($\beta = 1.26, p <.001$) as well as ventral striatum ($\beta = 0.69, p =.047$) and functional connectivity between CEN and amygdala ($\beta = 0.67, p =.047$) were positively associated with self-control success.

Using thresholded component maps as binary ROIs, we again found a significantly negative effect of NII on self-control performance ($\beta = -1.51, p <.001$), see Fig. 7f. Here, this effect was driven by a positive effect of SN-DMN connectivity ($\beta = 1.80, p <.001$) and a negative effect of SN-CEN functional connectivity ($\beta = - 0.91, p <.001$) on self-control performance. In addition, we found a positive effect of CEN-AMY ($\beta = 1.13, p <.01$) and DMN-VS ($\beta = 1.85, p <.001$), as well as a negative effect of CEN-VS ($\beta = - 0.90, p =.02$) functional connectivity on self-control performance.

Using 5 mm spheres, only the positive effect of DMN-AMY functional connectivity on self-control performance could be replicated ($\beta = 2.36, p <.001$).

Finally, using weighted sum time series to represent the ICA components, we once again found a negative effect of NII on self-control performance ($\beta = - 1.14, p <.001$), see Fig. 7g. Here, SN-CEN functional connectivity was negatively related to self-control performance ($\beta = -2.53, p <.001$) against our expectations. The effect of SN-DMN did not reach statistical significance but was descriptively positive ($\beta = 0.32, p =.25$). Finally, self-control performance was negatively predicted by DMN-AMY functional connectivity ($\beta = - 0.93, p =.02$) and positively by DMN-VS connectivity ($\beta = 2.10, p <.001$).

## Discussion

In this study, we analyzed resting-state functional connectivity correlates of task-based self-control performance in two datasets. Specifically, we focused on the functional connectivity of the salience network (SN) with central executive (CEN) and default mode network (DMN), respectively. Our hypothesis that stronger functional connectivity between SN and CEN and lower functional connectivity between SN and DMN would predict successful self-control exertion could not be confirmed. This hypothesis was initially derived from findings reported by Krönke et al.^13^ and the assumption that the SN plays a crucial role in switching between CEN and DMN activation. Indeed, both datasets and a joint analysis yielded evidence for the opposite direction of the expected effect.

In opposition to our main hypothesis, we found that a network interaction index quantifying the relative strength of SN-CEN compared to SN-DMN functional connectivity negatively predicted self-control performance across analysis pipelines and datasets. Notably, this index relies on the assumption that SN functional connectivity is generally positive with CEN and negative with DMN^13^. While we found this pattern at least descriptively in some of our analysis pipelines, there were also cases where this assumption was not met. Critically, however, we also found a negative effect of the network interaction index on self-control using anatomical network ROIs in dataset one, a case where SN was indeed positively coupled with CEN and negatively coupled with DMN. One possible reason for this effect directly contradicting our hypothesis is the fact that it is assumed that the functionality of the SN lies in flexibly switching between the activation of the DMN and the CEN to arbitrate between the processing of internal vs. external information^19^. The tasks used in both datasets analyzed here required decision-making in imaginary hypothetical scenarios (even though one choice was actually realized at the end of the experiment by Scholz et al.^23^). Hence, it seems plausible that internal, self-referential processing via the DMN might support task performance in these tasks. Indeed, there is some evidence for the role of DMN in self-referential goal-directed processing^24–26^ and specifically in episodic future thinking^43^ which has been linked to the successful exertion of self-control^44^. In other words, the detection of self-control conflict in more hypothetical scenarios might recruit regions of the DMN due to the self-referential nature of the task, whereas successful self-control conflict resolution in real-life situations (as measured by Krönke et al.^13^) might rely more strongly on CEN activation for processing external information (e.g., to inhibit tempting aspects of the environment such as the delicious smell of unhealthy food). In addition, Krönke et al.^13^ defined self-control failure in their real-life data as giving in to temptation after an attempt to resist the conflict-inducing desire was reportedly made. This measure excludes scenarios where no resistance was attempted (i.e., where desires were enacted volitionally despite a given conflict with a long-term goal). Our data and the data provided by the original authors of dataset two ^23^ cannot account for this particular aspect, so slightly different definitions of self-control have been applied across studies. However, we can only speculate about these potential explanations and future research is needed to replicate triple-network functional connectivity correlates of self-control success.

On the other hand, our main result was consistent across two datasets employing very different experimental measures of self-control, namely hypothetical real-life decision scenarios and food choices. Although one could argue that different cognitive processes might be required to support self-control across different domains, our result is in line with a body of empirical evidence showing that the fundamental neural mechanisms underlying self-controlled decision-making are surprisingly consistent across different domains. Across the domains of food choices^12,23^, real-life scenarios as in our study^11^, and physical exercise choices^45^, behavioral data have consistently supported the view of self-control as a value-based choice process based on the integration of anticipated action short- and long-term consequences. More crucially, this finding is consistently accompanied by the result that neural activation in the vmPFC reflects behavioral decision values and hence plays an important role in this valuation process. Notably, in an analysis of the task-based fMRI data corresponding to dataset one, we have replicated this seminal finding^37^, and the authors of dataset two observed the same pattern of results in their task-based functional data^23^. Hence, even though combining different paradigms might introduce some noise, it is plausible to assume that the fundamental cognitive processes of self-controlled decision-making are relatively domain-general.

In the exploratory joint analyses of both datasets, subcortical-cortical connectivity strength significantly predicted self-control success: Functional connectivity between both CEN and DMN with amygdala positively predicted self-control success in two out of four analyses pipelines, respectively. However, another analysis pipeline revealed the exact opposite trend for DMN-amygdala functional connectivity. As outlined in the introduction, CEN-amygdala functional connectivity strength has been negatively associated with regulatory functions in the past^31^ and we expected the same pattern in our results. While we cannot resolve this contradiction, the amygdala is part of the subcortical salience network. Hence, a positive association between CEN-amygdala connectivity and self-control would be in line with the hypothesis that SN-CEN functional connectivity positively relates to self-control success. Concerning DMN-amygdala functional connectivity, connectivity between amygdala and posterior cingulate cortex or precuneus (important hubs of the DMN) has been associated with successful emotion regulation^46,47^. Anatomically, the DMN also includes the vmPFC. Indeed, amygdala-vmPFC functional connectivity at rest has also been discussed in the context of emotion regulation^48^. Interestingly, while often studied separately, self-control and emotion regulation share important conceptual overlap (for a brief summary see elsewhere^49^) and it has been shown that the regulation of anticipated emotion is associated with self-control performance^50^. Further, a recent study has shown that neural activation during emotion regulation is predictive of self-control success^51^. Finally, we found DMN-ventral striatal connectivity to positively predict self-control success in three out of four analysis pipelines in the joint dataset. Task-based DMN-ventral striatal functional connectivity has been tied to reward processing^52^ and cortical regions of the DMN have been identified to represent an overlap between the brain’s default mode and valuation network, which also includes the ventral striatum^53^. Since both reward and valuation processes are central to the exertion of self-control, it seems plausible that connectivity between the DMN and corresponding regions is associated with better self-control performance. However, there are also contradictory findings in the literature^54^. Since all explanations presented here are post-hoc and purely speculative, future research is needed to systematically investigate the association between cortico-subcortical functional connectivity and the successful exertion of self-control.

Our study has several strengths that should not go unnoticed: first, we preregistered the analyses related to dataset one and included an exploratory alternative analysis pipeline trying to replicate previously reported results^13^ as closely as possible. Further, we included a second, independent dataset in our analysis to attempt further replication and the combined dataset yielded a substantial sample size (N = 200). The exploratory use of different analysis pipelines additionally generated some insight into the variability of overall connectivity patterns and behavioral correlates depending on a priori ROI selection and dataset. Despite great differences between both studies regarding the task at hand and the operationalization of self-control performance, results were rather consistent across both datasets.

However, there was still significant heterogeneity in the results of interest across datasets and analysis pipelines. This heterogeneity critically demonstrates how the selection of a priori network ROIs determines between-network functional connectivity patterns and functional connectivity correlates of task-based measures in a given sample. Across analysis pipelines the statistical significance and directionality of functional connectivity between SN and CEN or SN and DMN varied in this study. However, with regard to the interpretation of the computed network interaction index, the initial directionality of the identified connectivities is crucial: as outlined before, the logic of the computed index relies on the assumption of overall positive connectivity between SN and CEN and negative connectivity between SN and DMN. Different overall connectivity patterns (e.g., SN-CEN and SN-DMN both positive) or large individual variability in the directionality of between-network connectivity complicate the interpretation of network interaction indices and comparability across participants and studies. In addition, this variability poses the question of which ROI selection method reflects the networks of interest to this study best. Here, data-driven approaches might be more suitable to account for individual differences in functional network localization than predefined region of interest masks. However, even our data-driven ROI selection approaches still focused on using the same set of ROIs across all participants. There is some research suggesting that individualized ROIs might improve functional connectivity studies^55^. Future methodological research is needed to establish gold standards for ROI selection in resting-state functional connectivity analyses.

Further, the role of correlated and anti-correlated networks and the functional meaning of the sign of network correlation coefficients have been discussed extensively in the literature^41,56^. Here, it is worth mentioning that we did not conduct global signal regression as part of our data denoising strategy. Global signal regression has been questioned in functional resting-state connectivity analyses^41^, and, hence, is not part of the default procedure employed by the CONN toolbox^40^. In addition, the authors of the original study^13^ that we attempted to conceptually replicate here did not use this method either. However, the role of global signal is highly debated^57^ and treating global signal differently across studies might contribute to further methodological variability limiting the generalizability and replicability of past findings. The use of different analysis pipelines, denoising strategies, and ROI selection methods yielding heterogeneous connectivity patterns across studies complicates this discussion significantly and makes the interpretation of resting-state functional connectivity results across studies difficult. It is noteworthy, nevertheless, that our data yielded relatively consistent associations between the network interaction index and self-control performance across ROI selection methods, especially in dataset two, suggesting that different ROI selection methods still yielded a similar association between neural and behavioral data. Unexpectedly, however, this main result never reached statistical significance when using the 5 mm spheres used in the original study^13^. Potentially, these smaller ROIs are more susceptible to interindividual variability in precise functional network locations than the larger network ROIs or components and could consequently fail to capture the intended network dynamics more easily.

Finally, functional resting-state connectivity is assumed to reflect functional processes in the brain that are not only relevant at rest but also relate to active cognitive processes^58^ (an assumption that is critical for this work) and resting-state network connectivity patterns show critical alterations in patients with psychiatric disorders^27,59^. However, the use of resting-state functional connectivity analyses has been criticized recently: major criticisms concern sample sizes^60^, high noise and low reliability^61^, or question the use of studying resting-state connectivity to understand neural mechanisms of active cognition in general^62^. While we do not share the latter point of view, we agree that reported resting-state correlates of task-based measures must be interpreted with caution.

In addition to methodological considerations regarding resting-state functional connectivity analyses, it is also important to acknowledge that previous studies investigating resting-state correlates of self-control, as well as the two datasets we used in this study, critically differ in the choice of outcome measure quantifying self-control performance. While Krönke et al.^13^ assessed self-control in daily life using ecological momentary assessment, we used a task where participants made imaginary daily-life-like choices (e.g., decided whether they would stay at home to watch a movie despite having planned to go for a run) and Scholz et al.^23^ employed a food choice paradigm where participants made choices between food items with differing health and taste attributes. We cannot provide any evidence for the convergent validity of these measures, but self-control measures in general have been shown to have moderate convergent validity at best, with large heterogeneity depending on the tasks examined^63^ and laboratory tasks cannot capture the full complexity of self-controlled decision-making in real life. As discussed above, this might introduce some noise and consequently reduce comparability across studies. On the other hand, as also discussed above, there is convincing evidence for shared behavioral and neural mechanisms of self-control across different domains.

In conclusion, using a laboratory task in which participants made imaginary daily-life-like choices involving self-control conflicts, we could not replicate past research suggesting that resting-state functional integration of the salience network with the central executive network and functional disintegration of the salience network with the default mode network is predictive of the successful exertion of self-control. Instead, our results point towards the exact opposite effect, namely a negative effect of relative functional connectivity between salience and central executive vs. salience and default mode network on self-control performance, directly contradicting our main hypothesis. The same was true for a second dataset, where food choices were used as a measure of self-control. This indicates that self-controlled choices in laboratory tasks and in real-life temptation contexts may be partly mediated by different mechanisms. In particular, this result may point towards an important role of the default mode network in self-referential goal-directed processing when self-control decisions are made in hypothetical scenarios. We further showed that overall functional connectivity patterns and correlates of self-control vary significantly across datasets and analysis pipelines. Future research is needed to address our findings in greater detail: Dynamic connectivity analyses could contribute to a time-resolved understanding of the role of the salience network in switching between the central executive and the default mode network in the context of self-control. This aspect is of particular interest as research increasingly suggests that the precise temporal dynamics of network activations contribute to optimal cognitive functioning^64^. Finally, the role of cortico-subcortical functional connectivity in self-control needs to be studied more systematically.

## Data Availability

The aggregated data sets (indices of self-control success and between-network functional connectivity for each subject) generated and analyzed during the current study as well as analysis scripts used for determining brain-behavior correlations are available in the OSF repository, https://osf.io/d2h7y/files/osfstorage.
